# Effect of post-exercise ingestion of different molecular weight carbohydrate solutions. Part II: The incretin response

**DOI:** 10.1186/1550-2783-12-S1-P31

**Published:** 2015-09-21

**Authors:** Anthony J Anzalone, Anthony L Almada, Leighsa E Van Eck, Margaret T Jones, Andrew R Jagim, Joel B Mitchell, Meena Shah, Jonathan M Oliver

**Affiliations:** 1 Department of Kinesiology, Texas Christian University, Fort Worth, TX 76129, USA; 2 Vitargo Global Sciences, LLC, Dana Point, CA 92629, USA; 3 Health and Human Performance Division, George Mason University, Fairfax, VA 22030, USA; 4 Exercise & Sport Science Department, University of Wisconsin - La Crosse, La Crosse, WI 54601, USA

## Background

Gastric inhibitory peptide (GIP) and glucagon like peptide-1 (GLP-1), incretin hormones of the small intestine, are secreted in response to the presence of food in the lumen. Once released into circulation, these incretins stimulate beta cells to increase insulin secretion, accounting for at least 50% of total insulin secreted after glucose ingestion. Post-exercise ingestion of a high molecular weight (HMW) carbohydrate (CHO) solution has been shown to result in greater rates of muscle glycogen synthesis, which are attributed to the higher rates of gastric emptying, compared to a low molecular weight (LMW) CHO solution. However, no studies have examined the effect of post-exercise ingestion of CHO's of differing molecular weights on incretin response. Therefore, we sought to examine the difference in GIP and GLP-1 secretion after ingestion of HMW and LMW CHO solutions following a glycogen depleting exercise bout.

## Methods

Sixteen resistance trained men (mean ± SD; 23 ± 3 years; 176.7 ± 9.8 cm; 88.2 ± 8.6 kg; 12.1 ± 5.6 % fat) participated in this double-blind, placebo-controlled, randomized cross over study, which consisted of three testing sessions, each separated by one week. VO_2_ max (37.4 ± 4.3 ml·kg·min^-1^) was determined prior to testing session 1. In sessions 1-3, subjects completed a glycogen depleting cycling bout of 60 minutes at 70% VO_2_ max, followed by six, one-minute sprints at 120% VO_2_ max. Immediately post-exercise, subjects ingested a placebo (PLA), or a LMW or HMW CHO solution (10%) providing 1.2 kg· bw^-1 ^CHO, assigned randomly. Blood was sampled prior to ingestion and every ten minutes for 120 minutes post-ingestion. A two-factor repeated measures ANOVA was used to determine differences among treatments (p ≤ 0.05).

## Results

A time × treatment effect was observed in both GIP (p < 0.001) and GLP-1 (p < 0.001). Ingestion of both HMW and LMW solutions caused a sharp increase in GLP-1 and GIP, resulting in significantly higher values compared to those observed following ingestion of PLA. By 10 minutes both GIP (LMW, 146.7 ± 6.5 pg·mL^-1^; HMW, 129.7 ± 23.7 pg·mL^-1^) and GLP-1 (LMW, 13.1 ± 3.3 pg·mL^-1^; HMW, 13.2 ± 3.3 pg·mL^-1^) were higher following ingestion of LMW and HMW compared to PLA (GIP, 35.1 ± 6.1 pg·mL^-1^; p ≤ 0.004; GLP-1, 2.1 ± 0.5 pg·mL^-1^; p ≤ 0.001). GIP increased progressively and remained elevated for the entirety of blood sampling (120 minutes) in both CHO conditions. Changes in GLP-1 were almost immediate, resulting in a trend, whereby GLP-1 values were elevated above PLA immediately post-ingestion in both LMW and HMW (p = 0.089 and p = 0.087, respectively). GLP-1 peaked at 40 minutes following ingestion of LMW (27.9 ± 3.5 pg·mL^-1^) and HMW (28.5 ± 5.1 pg·mL^-1^), then began to decline, remaining above PLA until 120 minutes. No differences were observed between HMW and LMW GIP or GLP-1 at any time point.

## Conclusions

These data suggest ingestion of HMW and LMW solutions providing 1.2 kg· bw^-1 ^CHO result in similar responses in the gut hormones GIP and GLP-1. Further study is needed to determine incretin's effect on subsequent insulin secretion and glucose disposal.

